# Characterization of broiler poultry production system in Rwanda

**DOI:** 10.1007/s11250-016-1160-0

**Published:** 2016-10-11

**Authors:** Francis Mbuza, Rosine Manishimwe, Janvier Mahoro, Thomas Simbankabo, Kizito Nishimwe

**Affiliations:** 1 Centre for Livestock Research and Development (CLRD), Butare, Rwanda; 2 School of Animal Sciences and Veterinary Medicine, Uppsala, Sweden; 3 School of Food Science and Technology, College of Agriculture, Animal Sciences and Veterinary Medicine (CAVM), University of Rwanda, Butare, Rwanda

**Keywords:** Broiler poultry, Broiler sector, Performance parameters, Challenges, Rwanda

## Abstract

A study was conducted on 37 randomly selected broiler poultry farmers in Rwanda to characterize the production system using pre-tested semi-structured questionnaires. The data were processed in SPSS and presented as means, percentages and ranges in tables and text. All respondents kept Cobb breed and young stock was mainly (73 %) imported from abroad. The majority of respondents were males (68 %) and most farmers had attended only primary level of education (40.5 %). Most of the farms were in the peri-urban (48.6 %) and urban (37.8 %) areas and hired male youth (62.2 %) mainly aged 19–35 years. The majority of respondents (68 %) kept less than 500 birds per batch. Recordkeeping was well practiced (91.9 %) and (62.6 %) had permanent poultry houses and all farmers used deep litter system. Purchased feedstuffs were reportedly (92 %) mixed at farm level as the main feed resource. Maize bran was reported (97.06 %) the main, basal feedstuff. The mortality rates of chick and growers were 12.3 and 9.4 %, respectively. The slaughter age was reportedly 60 days with an average dressing percentage of 75.67 %. The main challenges reported were scarcity and unaffordability of quality feeds (59.5 %), lack of market access (45 %) and lack of credit (21 %). The farmers had various views on improving broiler production in Rwanda ranging from establishing feed processing industries 62.2 %, improving marketing facilities 35.1 %, increasing availability of day-old chick and access credit 27 %, to intensification of farmer training 16.2 %.

## Introduction

Livestock rearing is an important subsidiary occupation that supplements the income of smallholder farm families and rural households in most tropical developing countries. As indicated by Assa (2012), poultry production is the fastest growing component of global meat production, with developing and transitional countries assuming a leading role. The poultry sector all over the world is continuously growing due to increasing of human population, increase in purchasing power and urbanization. However poultry production in Africa is lagging behind that of other continent such us Asia (FAOSTAT 2014).

Poultry possess greater efficiency in converting feed into egg and meat compared to other livestock enterprises. Commercial poultry farms play an important role in meeting national protein supply (Jabir and Hague 2010) through the supply of eggs in addition to poultry meat. Commercial broiler production on the other hand provides mainly poultry meat as birds are raised solely for meat.

But in some African countries, broiler production is becoming important. For example in South Africa, broiler meat production occupies 35 % of all animal products (South Africa Department of Agriculture and Forestry and Fisheries 2012).

In comparison to other eastern Africa countries, poultry meat production in Rwanda is still very low. The total poultry population in 2012 was estimated (NISR 2013) to be 4688 million birds.

Rwanda is a country characterized by a high population density, where the population growth is estimated at 2.9 % per year (Minagri 2012). The poultry sector of Rwanda is one of the cluster areas for value chain development. Poultry are the most predominant in terms of numbers with an estimated population of 4.8 million birds (FAOSTAT 2014). Over 80 % of the small-scale farmers in Rwanda rear chicken, majority of which are indigenous (FAO 2009; NISR 2011).

In order to further alleviate poverty and insure food security in the long run, the efficient use of the available land through poultry and small livestock production could be one of the options for emphasis. The agricultural sector contributes over 40 % of the national Gross Domestic Product and almost 90 % of the export earnings. Livestock including poultry contributes 6 % of the GDP and about 30 % of the agricultural GDP. It therefore calls for an urgent need to increase broiler production at both household and commercial holdings in Rwanda. The Rwandan vision regarding the poultry industry (Minagri 2012) is to make the poultry industry a flagship of the Rwandan livestock in order to increase protein intake and address food insecurity in the country.

In developing, tropical countries like Rwanda, a number of factors are likely to constrain optimum broiler poultry production. In study on 13 factors likely to influence broiler production in Bangladesh (Ali and Hossain 2008), it was found that education, land possession, annual family income, training exposure, broiler farming experience, broiler farm size, capital in broiler farming and extension contact had significant positive relationships with broiler performance.

Due to the lack of grazing land in Rwanda, the scope for development of livestock industries based on large animals is limited. Broiler production is one of viable enterprises under such conditions. Broiler farming is likely to become popular with farmers due to short life cycle and low capital investment (Raha 2007). Chicken meat is acceptable by all sectors of consumers, irrespective of sex and religion.

Broiler can be produced within the least possible time compared to other meat-producing animals. However, broiler production in Rwanda is still low due to some problems during its development, as broiler farmers face a great many input problems. Successful broiler rearing depends on many inputs—such as availability of quality chicks, supply of quality feed, medicine and vaccine and other support services. Each of these factors is vital for profitable broiler farming.

In a recent study sponsored by the ministry of agriculture (Menagerie) of Rwanda (Minagri 2012), the poultry industry of Rwanda was assessed to develop the strategy and investment plan to strengthen the poultry industry in Rwanda within the period 2012–2017. The study concluded that there was great potential for improved poultry production in Rwanda for the local market and possible export to the accessible markets of Democratic Republic of Congo, Burundi and Congo Brazzaville. Whereas this study dwelt on value chains emphasizing marketing, processing and price, it ran short of assessing the production practices of emerging layer and broiler farmers. Unfortunately, little is known about the current situation of the broiler production industry in Rwanda. This study therefore aimed at characterizing the broiler poultry production systems in Rwanda to determine the current status of management, production and marketing practices, identifying the challenges faced by farmers so as to propose improvement and interventions.

## Material and methods

The study was done in the four provinces of Rwanda (eastern, northern, southern and western) in addition to Kigali City in the period of 2014–2015. The 37 respondents were chosen through a multistage sampling procedures at province, district and sector levels.

Due to time, financial resources and availability of technical staffs, the sampling rates at different stage were that all provinces were surveyed and 50 % of districts selected from each province except in Kigali City where all the districts were selected. Within the selected district, 10 % of the sectors were selected except in Kigali City where three sectors were selected from each district (Table 1).

**Table 1:** Selected district and sectors in the study areas

Province	Total districts	Selected districts (50 %)	Total sectors	Selected sectors 10 %
East	7	3.5 ≈ 4	95	10
West	7	3.5 ≈ 4	96	10
North	5	2.5 ≈ 3	89	9
South	8	4	101	10
Kigali City	3	3 (100 %)	35	9

All broiler poultry farmers in each selected sector were interviewed ending up with a total of 37 respondents. The local government staff at sector level guided a numerators to locate the broiler poultry farmers.

A pre-tested semi-structured questionnaire was used by pre-trained academic staff to gather the necessary data from identified broiler poultry farmers. The data were entered in the computer using the software SPSS 16.0 (2009) and processed.

## Results and discussion

### Distribution of respondents among the surveyed districts

The 37 respondents comprised of female 51.3 % and males 68 % as owners and managers of the broiler poultry farms. The education level of the owners was generally basic as 40.5 % of the respondents had only attend primary education while 31.5 % had attend secondary education.

Most farmers were from eastern and western provinces (10 farmers each), followed by Kigali City (9 farmers), and northern and southern provinces with 4 farmers each. Among the districts, the largest number of respondents were from Rubavu in western province which is known to have a high population of poultry farmers whereas Kayonza and Rwamagana in eastern province had only one farmer each. The large population of broiler poultry farmers in western province can be attributed to its proximity to Democratic Republic of Congo (DRC) where a ready market of broiler chicken prevails. Kigali City also had a high population of broiler farmers attributable to existence of super markets, hotels and big restaurants.

Most of the respondents (48.6 %) were from the peri-urban areas while 37.8 and 13.5 % were from the urban and rural areas, respectively. The location of broiler poultry farmers was mainly cross to feeder roads as 43.2 % were within 1 km, 37.8 % were within 1–2 km with only 20 % above 2 km from the feeder road. These results indicate that broiler farmers were aware of the importance of feeder road and urbanization for easy access to farm inputs and marketing of farm produce.

### Gender and education levels of managers

In most cases, managers of broiler poultry farms (75.7 %) were males and most of them had attained primary and secondary level of education. This shows some potential for improved broiler production as level of literacy and numeracy is required for effective management and production of broilers poultry. It is logical that large training exposure with high education and experience makes a farmer better able to do his job properly (Ali and Hossain 2008).

In addition, husbands (78.4 %) were responsible for management of funds accruing form the farm enterprise. The male age group of 19–35 years was mostly responsible for daily farm tasks such as cleaning 64.9 %, treatment 48.6 %, purchasing 48.6 %, selling 45.8 % and feeding 64.9 %. Overall, women of all age groups were rarely involved in broiler poultry farm activities.

In a study in western Kenya (Okitoi et al. 2007), ownership of rural poultry was found to be shared among the family members but was predominantly by women (63 %) and children (18 %). On the other hand, decision-making regarding selling, consumption and gifts to guests in rural poultry reflects plurality. All family members provided labour to a rural poultry production enterprise. Men and children mainly did construction of poultry sheds as women did cleaning, feeding and treatment of rural poultry. Women and children did most of the daily routines in rural poultry management. Men did occasional jobs that were cash requiring such as purchase of inputs and treatment of poultry using conventional drugs. Women did occasional sale of eggs. Women dominated the access and control of food and gifts to guests while men dominated cash and cultural benefits arising from poultry.

In conclusion, ownership of rural poultry and access to benefits was not exclusively the domain of women. Decision-making by women in the rural poultry production system was limited to non-cash-related decisions while cash-related decisions were made mostly by men. The family labour input into the rural poultry production system was a plurality.

### Breeding

Most of broiler poultry farmers (73 %) chose the rearing stock based on the performance (growth late), followed by survival traits, e.g. resistance to disease (Table 2) as cited by neighbouring farmers. The cost of the day-old chicks was also reported to be considered in breed choice criteria.

**Table 2:** Criteria of choice of breeding stock

Selection criteria	Percentage of farmers who use the criteria
Performance (carcass weight)	73 %
Cost	32.4 %
Resistance	13.5 %

Cobb breed was predominately the preferred breed by all broiler poultry farmers in the study. This breed has very high growth performance potential especial when fed on appropriate rations with proper amino acid regimes (Vieira et al. 2012).

The challenges therefore is whether the broiler farmers in Rwanda have the knowledge and capacity to ensure optimal management of such breeds. Improved exotic chickens produce higher number of eggs and more meat than the indigenous chicken breeds, but the tropical climate is a great challenge. They are not adapted to adverse environmental conditions, such as high temperature, disease and shortage of feed.

Importation (94.6 %) was the commonest mode of securing day-old chicks and most of the respondents (75 %) reported importation of day-old chicks from neighbouring Uganda. Whereas importation ensures access to high-performing genotypes from parents stocks abroad, it can be very costly to farmers as day-old chicks become expensive and often reach the farmers when they very tied. In this study, the average cost of day-old chicks was 800–1000 Ruff equivalent (1.14–1.42 USD per chick) at date of the study.

The average flock size per farm varied from province to province and district to district. Within a province, there was great variation between the minimum and maximum flock size which is a feature indicative of an evolution process from small substance farms to medium semi-commercial and commercial production farms.

It is noteworthy that most broiler poultry farmers (68 %) kept less than 500 birds per batch (Table 3) and only a few (22 %) kept more than 1000 birds per batch. This could be attributed to minimal availability of space, funds, day-old chicks or other resources to enable perches of a large number of chicks in a single batch. With such small flocks, it is doubtable weather farmers can make sufficient profit margin for social economic developments. In Bangladesh (Kawsar et al. 2013), profitability of small-scale broiler farming was found to depend on several factors, of which FCR had the highest impact (−0.711) and profitability increased with the size of the farm.

**Table 3:** Number of reared birds per batch

Range of the number/batch reared birds	Frequency	Percentage
Less than 100 birds	8	22 %
100–500 birds	17	46 %
600–1000 birds	4	11 %
More than 1000 birds	8	22 %
Total	37	100 %

These results are similar to those of a study done in Nigeria (Emaikwu et al. 2011), where it was found that majority of the respondents (83 %) had broilers birds ranging from 100 to 500 followed by 13 % in the range of 501 to 1000 broilers while 4 % had a flock size in the range of 1001 to 1500 broilers.

### Farmer training in broiler poultry management

Broiler poultry farmers lacked extension services as only 35 % of the respondents reported having previous training in poultry management. Extension services are not only important to facilitate adoption of an innovation but also a necessary tool for success due to ease of implementation of innovation for profitability (Ali and Hossain 2008).

### Categories of labour on broiler poultry farms

Family labour was found to play a significantly low role on the surveyed broiler farms as majority (62.2 %) of the farms used hired labourers on full time or occasionally, only 21.6 % were using family workers and 16.2 % were using hired and family labours concurrently.

This resembles the situation cited in Kaduna state, Nigeria (Emaikwu et al. 2011) were the majority of the respondents (87 %) employed both family and hired labour in their production process while 13 % of them employed purely family labourers. None of the respondents in Nigeria exclusively employed hired labour.

### Keeping of farm records

Record keeping was a common practice as broiler poultry (91.9 %) farmers reported keeping records especially those of feed usage. The records were most reportedly kept in farm record books (56.8 %), soft copies (17 %) and the rest on loose papers, wall and human memory.

### Housing

Permanent poultry promises were gaining ground as 63 % of broiler poultry farmers had constructed permanent structures (concrete floor, brick walls and iron sheet roofing). It was also noted that most of the poultry houses (91.9 %) were correctly oriented in the east-west direction and their distance was always less than 100 m from homestead. Most farmers however were not well conversant with the required levels of ventilation in poultry houses as only 5.4 % had poultry houses with the required ventilation levels of over 40 % of the wall area.

### Stocking rate in broiler poultry pens

Whereas the recommended average area per chick less than 4 weeks is 0.01–0.03 and 0.07 m^2^ for growers (Arbor 2009), the results showed that most broiler farms (46.8 %) were understocking. The situation was the same for other age groups of more than 4 weeks as most farmers (91.4 %) were providing space more than 0.05 m^2^ per bird. Such excessive space allowance per bird may be wasteful and counterproductive as birds spend more energy meandering and there is loss of efficiency due to space underutilization.

### Type of material used in the deep litter system

Most of all assessed broiler farms (94.6 %) in the study were using the deep litter system and saw dust was the most used material for litter (91.5 %). This results is similar to that found in Ghana (Anang et al. 2013) where the method of rearing the birds was the all-in all-out method. Thus, birds were kept in the same housing from day-old till they were disposed of farmers using the deep litter system because it was cheaper and easier to operate compared to the battery cage system.

### Feedstuffs, feeds and feeding

The results showed that majority of broiler poultry (92 %) farmers just bought feedstuffs mainly from commercial supplies and mixed the feeds at farm level. Only 8 % of broiler farmers purchased premixed commercial feeds. Maize bran was the main basal feedstuff used (97.06 %) in farm made broiler poultry rations followed by rice bran (35.29 %) and wheat bran (17.65 %).

With regard to protein sources, cotton seed cake and fish meal (79.41 %) were the most commonly used followed by soy bean meal (67 %); bone meal was most reported as sources of minerals (70.59 %) followed by lake shell (29.41 %).

It is notable that most of the feedstuffs such as cotton seed cake, fish meal, lake shells and others were being imported since the raw materials are not produced in Rwanda or are produced in very small quantities. Over dependence on importation of feedstuffs is likely to increase the cost of the final feeding rations and thus the need for encouraging the use local available possible substitute of the imported feedstuffs evil leguminous tree forage can substitute imported cotton seed cake.

USAID (2008) cited by Ali and Hossain (2008) reported that high price of feed was a major problem in Bangladesh due to raw materials (maize, wheat and soya meal) being imported from other countries. In Ghana (Anang et al. 2013), feed cost was also reported to be the most important cost item followed by chick cost and labour cost. Together, these accounted for 90 % of the total cost of broiler production.

In Rwanda there is growing competition between livestock and humans for maize and its by-products. However, the maize yield per hectare is too low to meet the needs of the ever growing human and livestock population. This situation is enough to justify the exploration of the usefulness of other carbohydrate-rich feedstuffs as maize replacers in feed formulations. The possible maize replacers included cereal and by-product, roots and tubers, fruits and by-product, etc.

In most developing countries like Rwanda, the most commonly used protein sources for animal nutrition are by-products of oil seed and fish meal, all of which are very expensive and scarce. In a recent study in Zimbabwe (Gadzirayi et al. 2012), it was found that dried forage leaf of *Moringa oliefera* as protein supplement in broiler diet at 25 % inclusion level produces broiler of similar 274 weight and growth late compare to those fed under conventional commercial feeds. In this 275 way, poultry rations will become more cost effective than hitherto.

### Daily quantity of feed and frequency of feeding for the different age categories

The daily quantity of feed given to broiler birds at different age categories (Table 4) was general at the top end of the recommended range according to Arbor 2009 and the frequency of feeding was less ad libitum feeding.

**Table 4:** Daily quantity (gs/per bird) and frequency of feeding in broiler farms as compared to the expected standards (Arbor 2009)

	Mean	Standard deviation	Recommended range
			(Arbor 2009)
Quantity of feed DOC (g/day/chick)	35.2	40.7	12–35 g/chick/day
Frequency of feeding DOC/day	2.7	0.86	Adlib
Quantity of feed 4–8 weeks chicken (g/day/chick)	135.0	47.5	35–135 g/bird/day
Frequency of feeding 4–8 weeks chicken/day	2.5	0.70	Adlib

### Health management on broiler poultry farms

The majority of the respondents (83.8 %) reported read access of veterinary services whenever required and most of them (58 %) preferred private veterinary service providers. Biosecurity measures such as antiseptic foot bath were available at 51.4 % of broiler poultry farms. However, most of the farmers (62.2 %) did not properly clean the poultry premises as they never disinfected the pens before introducing new batches of day-old chick. This could partly explain the reported high mortality rates (14 %) of broiler chicks before 4 weeks while after 4 weeks it drops to 9 %. In Ghana (Anang et al. 2013), a study showed that mortality rate of broiler birds averaged 17 % and the reliability of the source of day-old chicks was found to be important in ensuring low mortality rates.

### Production parameters on broiler poultry farms

The results revealed inefficiency in the production systems as most of the parameters were generally lower than the recommended levels (Table 5). The reported mean age at slaughter of 60 days ± 14.9 is likely to be uneconomic to the farm. The results revealed a dressing percentage (75.67 % ± 16.5) which is inferior to that reported by Kalio and Okafor (2012) in Nigeria, where the dressing percentage was 78.41 % in a semi-intensive rearing system and 81.78 % in an intensive rearing system. The high age at slaughter indicates inefficiency as it leads to unnecessary expenditure on feeds and other management costs, and it delays restocking.

**Table 5:** Production parameters of broiler poultry on surveyed farms

	Mean	Standard deviation
Age at slaughter (days)	60	14.9
Average live weight of broilers at slaughter (kg)	2.4	0.76
Average carcass weight (kg)	1.98	0.66
Dressing percentage (%)	75.7	16.5

### Broiler marketing

Three modes of marketing were identified. Contract sale was reported as the most common mode of selling for the birds with less than 8 weeks of age (48.2 %). For chickens with more than 8 weeks of age, direct sale at the market place was reported as the most common mode (41.4 %). Direct sale at the farm gate was least practice (24.1 %) for both birds’ categories below and above 8 weeks.

### Challenges reported by broiler poultry farmers

Broiler poultry farmers mentioned many challenges of which lack of quality feeds (59.5 %) and poor access to markets (45.9 %) were most cited. Prevalence of poultry diseases (32.4 %) and lack of training on modern poultry production practices (8.1 %) were also cited as challenges. These may indicate poor service provision from private companies that supply feeds, credits, etc. These results resemble those recently found in Ethiopia (Yemane et al. 2016) where the high price of feed, shortage of land, unavailability of pullets in time, high cost of pullets, feed quality, shortage of water, unavailability of feed in the nearby area, marketing difficulties during selling, health problem, lack of access to credit and inadequate training were the major constraints in small-scale intensive urban poultry production.

In Ghana (Anang et al. 2013), inadequate finance was identified by broiler poultry farmers as the most critical constraint limiting farmers’ ability to carry out management practices like feeding and diseases control as well as the purchase of day-old chicks. The broiler poultry farmers proposed suggestions for improving poultry production in Rwanda (Table 6) with the major one being the establishment of feed processing industries.

**Table 6:** Suggestions from respondents to improve broiler production

Suggestion	Percentage of famers that stated the proposition
Establish feed processing industries	62.2 %
Increase availability of day-old chicks	27 %
Improvement of market facilities	35.1 %
Increase access to credit facilities	27 %
Training	16.2 %

### Recommendations

The entire poultry value chain of Rwanda require special attention in the areas of research extension and developments to propel it to international standards. Poultry farmers need training in all aspects of production and management such as feeding, breeding, housing, health and entrepreneurship. Special attention should be put on developing the national animal feeds industry using the supply chain approach. Alternative sources of poultry feedstuffs should be identified, evaluated and commercialized. Poultry farmers should also be encourage to form production and marketing farmer groups or cooperatives. Adaptive research is required for testing and evaluating improved technologies from abroad to adapt them to the Rwandan situation. Effective linkages should be developed and strengthen between poultry producers and financial institutions to enable easy access to credit facilities.

A national poultry policy should be in place to improve the organization of production and marketing, allowing increase in stability and security of poultry output throughout the year. In addition, efforts should be taken to ensure safety standards of poultry meat for human consumption. Experts from the government, research institutes, universities, NGOs and other relevant sectors need to work in a collaborative manner in order to allow sustainable production and fight challenges jointly whenever they arise. Corresponding attention to research and development will allow the development of poultry sector in Rwanda. As government funding is limited, poultry industries need to come forward either to establish their research facilities or to provide funds to universities and research institutes in order to undertake research works of national and international importance.
